# An Epidemic of Trichiniasis on the Jordan

**Published:** 1884-01

**Authors:** 


AN EPIDEMIC OF TRICHINIASIS ON THE JORDAN, (Virchows Archiv, vol. 83, p. 553).—The question of trichiniasis is one of such great importance that every communication of its distribution among animals of an unusual nature is of great value; this case is especially instructive as an American observer, Dr. J. Payne of New Orleans, has asserted that among the hogs which he examined *those that were cornfed* were mostly infected.

In the case we are about to notice, the disease in human beings was due to a "wild hog caught in the swamps of "El Hulah" on the 25th November, 1880. It was a large boar, and its flesh was pronounced to be sweet, fat, and healthy. A great many persons of the village eat the meat partly half cooked, not one of whom escaped the disease. The head of the boar was sent to a family as a present; *it was cooked three times* before any of these partook of it; *and no one was sick therefrom.*

All those that ate the meat were apparently as well as usual until the disease became manifest which was in the second week from the time they had eaten the meat, though in some cases no disturbance became apparent until the third week. One person was taken with emesis shortly after eating; in this case the disease assumed a very mild form. Another had cooked the meat very thoroughly and was only slightly attacked. The chief phenomena from the third to the fifth week were aedema of the face and extremities; acute muscle pains; variable degree of fever, and itching over the whole body. The aedema was in some cases very extreme and extended over the whole body. Young persons suffered less than aged.

Convalesence followed very slowly and was accompanied with much pain and great weakness.

Number of persons diseased:

| | |
|---|---|
| Males | 124 |
| Females | 103 |
| Children | 35 |
| Total | 262 |
| Died | 6 |

